# Local adaptation to seasonal cues at the fronts of two parallel, climate‐induced butterfly range expansions

**DOI:** 10.1111/ele.14085

**Published:** 2022-08-15

**Authors:** Mats Ittonen, Alexandra Hagelin, Christer Wiklund, Karl Gotthard

**Affiliations:** ^1^ Department of Zoology Stockholm University Stockholm Sweden; ^2^ Bolin Centre for Climate Research Stockholm University Stockholm Sweden

**Keywords:** climate change, diapause, Lepidoptera: Nymphalidae, phenotypic plasticity, photoperiodism, reaction norm, seasonal adaptation

## Abstract

Climate change allows species to expand polewards, but non‐changing environmental features may limit expansions. Daylength is unaffected by climate and drives life cycle timing in many animals and plants. Because daylength varies over latitudes, poleward‐expanding populations must adapt to new daylength conditions. We studied local adaptation to daylength in the butterfly *Lasiommata megera*, which is expanding northwards along several routes in Europe. Using common garden laboratory experiments with controlled daylengths, we compared diapause induction between populations from the southern‐Swedish core range and recently established marginal populations from two independent expansion fronts in Sweden. Caterpillars from the northern populations entered diapause in clearly longer daylengths than those from southern populations, with the exception of caterpillars from one geographically isolated population. The northern populations have repeatedly and rapidly adapted to their local daylengths, indicating that the common use of daylength as seasonal cue need not strongly limit climate‐induced insect range expansions.

## Graphical abstract

Many species that expand polewards as a result of climate change need to adapt to latitudinal differences in daylength because daylength guides life history decisions. We describe two parallel northward range expansions of a butterfly in Sweden and show experimentally that range margin populations at both expansion fronts have adapted locally to daylength. This has happened despite theory suggesting constraints to local adaptation at range margins and implies that the need to evolve new responses to daylength cues need not hinder range‐expansions.

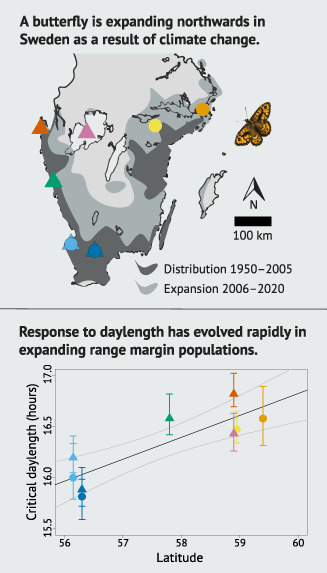

## INTRODUCTION

Climate change leads to poleward shifts in species ranges and changes in phenology, the timing of annual life cycles (Chen et al., 2011; Parmesan, 2006; Parmesan & Yohe, 2003). Changes in distribution and phenology are often simultaneous (Grevstad & Coop, 2015; Hällfors et al., 2021), but the evolutionary outcomes of their interplay are not well known (Macgregor et al., 2019). Although poleward‐moving populations may track a warming climate, they may still need to adapt to unfamiliar seasonal rhythms and environmental cues for life cycle timing. Latitudinal variation in daylength is particularly important because daylength guides the phenology of numerous organisms and does not follow changes in climate (Forrest, 2016; Saikkonen et al., 2012). Unfamiliar seasonal cues can lead to mismatches between phenology and the environment, making range‐expanding populations likely to face selection for local adaptation of life cycle timing, especially in areas with distinct seasons (Bradshaw & Holzapfel, 2007). Understanding the potential for adaptive evolution in range‐expanding populations is crucial for predicting future ranges of species, not least species under conservational concern, disease vectors and agricultural and forest pests (Diamond, 2018; Forrest, 2016; Mills et al., 2010; Saikkonen et al., 2012).

Daylength drives the timing of life‐history events in a vast range of animals and plants (Nelson et al., 2009) and induces, for example, dormancy, migration, and preparation for reproduction (Bradshaw & Holzapfel, 2007; Nelson et al., 2009). Daylength reliably mirrors seasonal variation (days lengthen from winter solstice to summer solstice, after which they shorten again), and this predictability allows animals and plants to prepare in advance before seasons change. But besides varying over the year, daylengths also differ among latitudes. Populations that expand across latitudes may thus encounter strong natural selection for altering their responses to daylength. Because the use of daylength to guide seasonal decisions is so widespread, latitudinal differences in daylength can profoundly affect the distribution of species, if responses to daylength do not evolve quickly (Bradshaw & Holzapfel, 2007; Saikkonen et al., 2012).

Range expansions are led by populations at the edges of species' ranges. Such range margin populations may not easily adapt locally because they can accumulate deleterious mutations (Kawecki, 2008; Peischl et al., 2013, 2015; Sexton et al., 2009) and experience serial founder effects during their expansion (Excoffier et al., 2009; Hewitt, 1996). Moreover, although gene flow from core range populations increases genetic variation at range margins, excessive gene flow can flood marginal populations with alleles that are locally adapted to the core range but maladaptive at the range margin, a phenomenon called gene swamping (Bridle & Vines, 2006; Hoffmann & Blows, 1994; Kawecki, 2000; Lenormand, 2002). Despite these potential difficulties, several rapidly expanding invasive species display latitudinal clines in adaptive traits (Colautti et al., 2009). Such clines are consistent with adaptive evolution during range expansions that have advanced as fast as contemporary climate‐driven expansions of native species. However, constraints on evolution may be stronger in native species than in invasive species. Native populations need to cope with natural enemies, may face weaker selection than invasive populations and often have smaller population sizes and lower population growth rates than invasive populations (Colautti & Lau, 2015; Moran & Alexander, 2014). Further, invasive species can be expected to be unusually good at spreading and adapting, given that they have become invasive in the first place (Moran & Alexander, 2014). Demonstrations of local adaptation during contemporary climate‐driven range expansions of native species are scarce, but include advanced phenology in a plant (Lustenhouwer et al., 2018), changed food plant preference in a butterfly (Bridle et al., 2013; Buckley & Bridle, 2014) and improved cold tolerance in a damselfly (Lancaster et al., 2015).

Climate‐induced range expansions are particularly well‐recorded in insects (Hickling et al., 2006; Parmesan et al., 1999). In temperate climates, daylength serves as the main environmental cue for timing diapause, a state of seasonal dormancy helping insects survive winter and time reproduction to the summer (Denlinger, 2022). The timing of diapause is crucial in multivoltine populations (populations breeding more than one adult generation per growth season), where offspring born early in the growth season develop directly into adults, but later offspring enter diapause. Entering diapause too early in the summer is maladaptive because opportunities to reproduce are missed, and reproduction may fail completely if attempted too late in the autumn (Kerr et al., 2020; Van Dyck et al., 2015). Thus, it is unsurprising that local adaptation of responses to daylength is known in numerous insect species (Bean et al., 2012; Bradshaw & Holzapfel, 2007; Lindestad et al., 2019; Merckx et al., 2021; Tauber et al., 1986, pp. 201–207; Urbanski et al., 2012). The critical daylength (the daylength that induces diapause in half of a population) is, within a species, usually longer at high latitudes than at low latitudes (Bradshaw & Holzapfel, 2007; Danilevsky, 1965). This pattern stems from high‐latitude summers ending with long days—summers are short and summer days long at high latitudes. However, whether and how fast such local adaptation can evolve in range‐expanding populations is unclear. Local adaptation to daylength has evolved rapidly during range expansion of an invasive mosquito (Urbanski et al., 2012), but has not been shown in native species expanding in response to climate change (but see Lustenhouwer et al., 2018, who suggest daylength as a potential driver of earlier flowering in northward‐expanding populations of a shrub).

We studied local adaptation to diapause induction cues along two independent and parallel northern range expansions of the Satyrine butterfly *Lasiommata megera* (Linnaeus). Using citizen science data, we show that the species has expanded from southern Sweden northwards along both the eastern and the western coasts of Sweden in the years 2001–2020. The range expansion coincides with regional climate warming. Gene flow between the eastern and western expansion fronts is unlikely because *L. megera* is absent from central inland Sweden (Eliasson, 2005; Nordström, 1955; citizen science data presented in this study). Further, the species' latitudinal span and the timing of the range expansion are similar in the east and the west, which allows testing parallel evolution of response to daylength along the replicated range expansions. If natural selection for longer critical daylengths has been strong and the northern range margin populations have been genetically diverse enough, northern populations at both expansion fronts should enter diapause in longer daylengths than southern populations do. Alternatively, the northern populations may not have had sufficient time or genetic variation for local adaptations to evolve, or gene swamping from core range populations may have prevented local adaptation.

Using common garden laboratory experiments with populations from along both parallel range expansions, we demonstrate that the butterflies, except for those in an isolated island population, are locally adapted to the daylengths that they experience. In line with the adaptive prediction, northern populations entered diapause in longer daylengths than southern ones.

## MATERIAL AND METHODS

### Study species

*Lasiommata megera* is widespread in the Palearctic and has its northernmost range margin in Fennoscandia (Belyaev, 2020; GBIF.org, 2021a; Higgins & Hargreaves, 1983). This butterfly inhabits seashores, dry meadows and other grass‐dominated open habitats. Adults bask on stones and stone walls, and their fondness of the latter is reflected in the common name, wall brown. Food plant preferences in the wild are not known, but *L. megera* caterpillars readily feed on *Festuca ovina* L. (sheep fescue), *Dactylis glomerata* L. (cocksfoot or orchard grass) and several species in the genus *Poa* L. in laboratory conditions. Swedish *L. megera* populations are mostly bivoltine (breed two adult generations per year), but may have a partial third generation in warm years. Unlike most temperate butterflies, *L. megera* is not known to shift to univoltinism (one generation per year) in the northernmost parts of its range. *Lasiommata megera* caterpillars typically enter diapause in their third (penultimate) larval instar and are sensitive to diapause induction cues until this stage.

### Analysis of the range expansion

We studied the recent range expansion of *L. megera* using citizen science data from Scandinavia, Finland, and the Baltic states. Many more occurrence records are available for the 2000s than for the 1900s (Table S1), which we corrected for by identifying well‐recorded areas. In QGIS 3.16 (QGIS.org, 2021), we mapped all observations of species in the superfamily Papilionoidea reported in the time periods 1901–2000, 2001–2010, and 2011–2020 onto a map grid of 20 × 20 km squares. We then calculated the numbers of Papilionoidea observations, Papilionoidea species, and *L. megera* occurrences for each square during each time period. The increase in number of reported species with number of occurrence records slows down at about 100 occurrence records within a 20 × 20 km squares (Figure S1). Thus, all squares with at least 100 occurrence records of Papilionoidea butterflies in each of the three time periods were considered well‐recorded.

We analysed the Scandinavian range expansion (not including the few, scattered Finnish data points) by comparing the latitudes of the 10 northernmost *L. megera* observations among time periods. We did this separately for the eastern and western range expansions and divided Sweden into an eastern and a western part at 14.31°E. Using linear models with latitude as response variable and time period as explanatory variable, we tested whether the northern range margins have shifted northwards. Further, we tested whether *L. megera* has expanded from warmer coastal areas towards the cooler inland. We did this by calculating the distance to the nearest coastline from the centres of all 20 × 20 km squares using the *Distance to nearest hub* algorithm in QGIS, and, with a linear model, analysing the relationship between time period and the ln‐transformed distance to coastline from the 10 *L. megera*‐occupied squares furthest inland. All three analyses included all squares with *L. megera* observations for 1901–2000, but only well‐recorded squares for the later time periods. Our tests were thus conservative and resilient to the low reporting activity for the earliest time period.

We downloaded the Swedish, Danish, Estonian, Latvian, and Lithuanian species occurrence data from the Global Biodiversity Information Facility (GBIF.org, 2021b); the Norwegian data from Artskart.no (Artskart.artsdatabanken.no, 2021) and the Finnish data from the Finnish Biodiversity Information Facility/FinBIF (2021).

### Diapause induction experiments

We performed two separate common garden laboratory experiments. In one—the eastern cline experiment—we compared diapause induction thresholds of *L. megera* caterpillars from southern Sweden (a regional range core) with those of caterpillars from populations close to the species' north‐eastern range margin. In the other—the western cline experiment—we compared the same southern populations with populations from the north‐western range margin.

**FIGURE 1:**
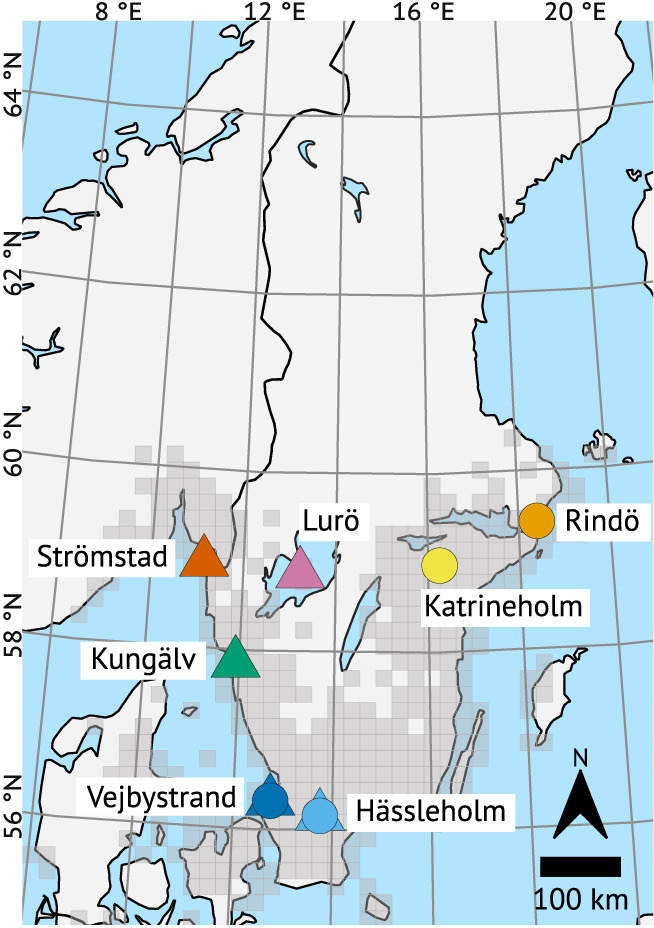
Our field collecting sites, later referred to as populations. Circles show the populations used in the eastern cline experiment, and triangles show the populations in the western cline experiment. We used Hässleholm and Vejbystrand in both experiments. The grey shading outlines the current range of *Lasiommata megera* in Scandinavia and Finland. This range is based on information from citizen science databases for the years 2011–2020.

In the eastern cline experiment, we used F2 offspring of mated females collected in July and August 2018 from two areas (henceforth, ‘populations’) in the southern (Hässleholm and Vejbystrand), and two populations in the north‐eastern (Katrineholm, Rindö) parts of the Swedish *L. megera* range (Figure 1; see Table S2 for coordinates and sizes of sampling areas). The offspring of the wild‐caught females fed on *Dactylis glomerata* in rearing cages in diapause‐inducing conditions (17°C and a photoperiod of 12 h of light and 12 h of darkness) until being moved outside in Stockholm (59.4°N, 18.1°E) into natural light and temperature conditions for the winter. In spring 2019, we fed the caterpillars with fresh *D. glomerata* and let them develop into adults. These adults mated in cages and laid eggs on *Festuca ovina*. The eggs developed in 17°C and a photoperiod of 12 h of light and 12 h of darkness until caterpillars, which we used in the experiment, emerged.

In the western cline experiment, we used F1 offspring of mated females collected in August 2020 from the same southern populations (Hässleholm and Vejbystrand) that we used in the eastern cline experiment, one intermediate‐latitude population (Kungälv) on the west coast, and two populations (Lurö and Strömstad) in the north‐western Swedish *L. megera* range (Figure 1; Table S2). Eggs of the wild‐caught females developed in various temperatures (10–27°C), which synchronised hatching and aided experimental logistics.

In both experiments, we placed 0‐ to 2‐day‐old caterpillars individually into 0.5 L rearing jars with *Poa annua* L. (annual meadow grass). Through a hole in the rearing jar, the grass reached another jar with water–fertiliser solution. We assigned the jars to daylength and temperature treatments maintained in climate cabinets (one cabinet per treatment) in such a way that offspring from different females were equally divided among treatments. We used climate cabinet models KB8400‐L and—for one treatment in the eastern cline experiment—KBP6395‐L, Termaks, Bergen, Norway. We replaced the grass whenever it looked dry or was almost completely consumed by the caterpillar.

The eastern cline experiment included eight climate cabinet treatments: four daylengths (14.5 h—that is, 14.5 h of light and 9.5 h of darkness—15.5 h, 16.5 h, and 17.5 h) crossed by two temperatures (16 and 22°C). With the two temperatures, we tested whether temperature affects diapause induction in *L. megera*. In the western cline experiment, we used only one temperature, 16°C, but added a fifth daylength treatment, 16.0 h, to improve the resolution of the experiment. Measured mean temperatures differed somewhat from the set temperatures and ranged from 15.1 to 15.8°C in the eastern cline 16°C treatments, from 16.0 to 16.7°C in the western cline 16°C treatments, and from 21.6 to 22.3°C in the eastern cline 22°C treatments. We report all mean temperatures and their standard deviations in Table S3. Sample sizes after mortality (which was 5.3% in the eastern cline experiment and 11.8% in the western cline experiment) were, per treatment, between 8 and 38 in the eastern cline experiment and between 14 and 19 in the western cline experiment. We report exact sample sizes along with numbers of families and mortality per treatment and population in Tables S4 and S5.

We weighed caterpillars several times to see how the weights of diapausing and non‐diapausing individuals diverge; non‐diapausing caterpillars are known to feed and grow quickly (Carle, 1969; Gotthard, 2008), but diapausing individuals stop growing in their diapausing life stage. We recorded pupation dates of non‐diapausing individuals and scored all individuals that had not pupated before the 55th day of the 16°C treatments and the 33rd day of the 22°C treatments as diapausing. These thresholds are based on the growth curves (obtained from the weight data) shown in Figure S2, and few individuals could reasonably be interpreted both as diapausing and non‐diapausing. To test how the chosen thresholds affect our results, we also analysed our data with alternative thresholds: pupation before the 49th or 61st day in the 16°C treatments and before the 29th or 37th day in the 22°C treatments.

We analysed our diapause induction data using generalised linear mixed‐effects models with binomial error distributions and logit link functions. The binary response variable was diapause or non‐diapause development. The explanatory variables were daylength (treated as a continuous variable), population, the interaction between daylength and population, and—for the eastern cline experiment—temperature and its interaction with population. Family was included as a random effect to account for the non‐independence of offspring from the same mother. We analysed the interaction between temperature and population because it tests whether populations differ in how much temperature modifies critical daylength; such local adaptation has been shown in a moth (Gomi, 1997). An interaction between population and daylength, in turn, could signal divergent selection on critical daylength in some populations. Such selection would be expected if some mainly bivoltine populations were partially univoltine or partially trivoltine. We excluded the interaction between temperature and daylength because its implications cannot be distinguished from those of the main effect of temperature in experiments with few daylength treatments when temperature matters only near the critical daylength. We tested the significance of the fixed effects using likelihood ratio tests, which we based on Type II sums of squares as no interactions were significant. We compared the populations pairwise using Tukey tests on models without interaction terms. To test whether the variance of the random effect deviates from zero, we performed likelihood ratio tests and divided the obtained p‐values by two to adjust for the conservativeness of likelihood ratio tests against boundaries (Pinheiro & Bates, 2000).

We estimated critical daylengths from models that we fitted separately for the different temperature treatments and that thus included only daylength and population as explanatory variables, as well as family as a random effect. We obtained the estimates (model estimates of the daylengths for which the probability of entering diapause is 0.5) by dividing each population‐specific intercept with the additive inverse of the slope of daylength explaining diapause. We bootstrapped 95% confidence intervals for critical daylengths as described in Lindestad et al. (2019).

## RESULTS

### Range expansion

**FIGURE 2:**
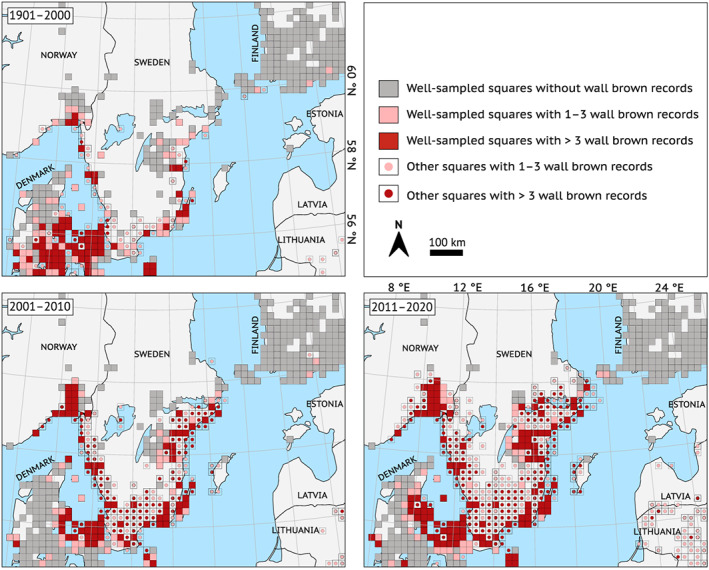
The range of *Lasiommata megera* in Scandinavia, Finland, and the Baltic states in the time periods 1901–2000 (top left panel), 2001–2010 (bottom left panel) and 2011–2020 (bottom right panel). Filled squares are well‐recorded (more than 100 records of Papilionoidea butterflies in each time period), circles show other (that is, not well‐recorded) squares where *L. megera* observations have been recorded, and colours indicate how many records there have been in each time period (see legend). Squares with light‐red symbols have only 1–3 *L. megera* records and should be interpreted with caution, as some of them may show stray individuals, very sparse populations, or misidentifications. Squares that neither are well‐recorded nor have *L. megera* observations are not shown. Note that the Lurö population (see Figure 1 for location) shows up on the 1901–2000 map, but that the first reported sighting is from the very last year of that time period.

**FIGURE 3:**
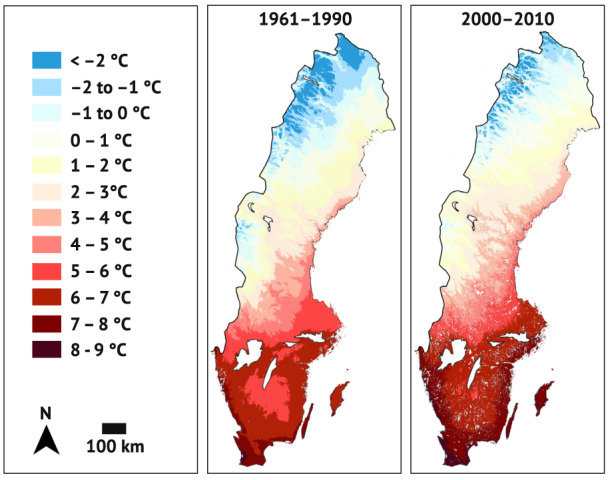
Annual mean temperatures in Sweden for the time periods 1961–1990 (left) and 2000–2010 (right). The changes in the southernmost third of Sweden correspond well with the range expansion of *Lasiommata megera* (Figure 2). We downloaded the data over 1961–2000 from the WorldClim database (Fick & Hijmans, 2017). The map over 2000–2010 was made with data from Meineri and Hylander (2016a, 2016b). The spatial resolution is 30 s (approximately 1 km^2^) for 1961–1990, and 50 m for 2000–2010.

**FIGURE 4:**
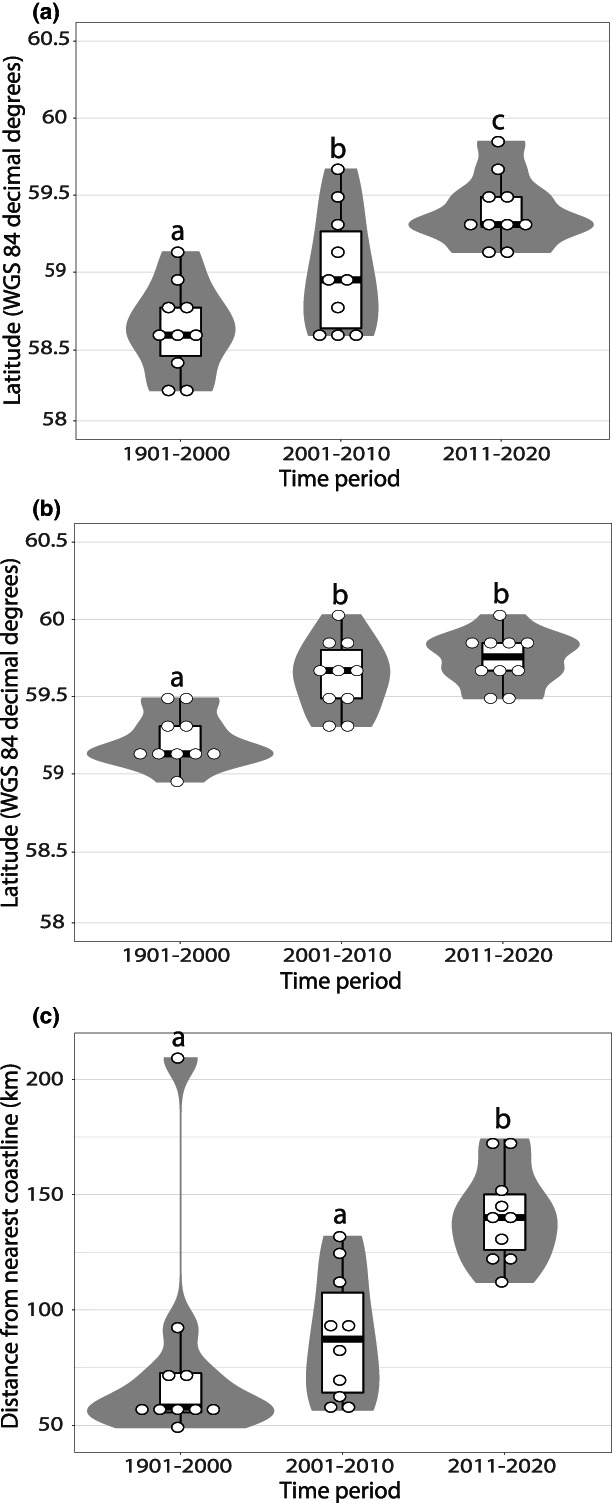
The locations of the 20 × 20 km squares with *Lasiommata megera* observations furthest north and inland in Sweden and Norway in each time period: (a) the 10 northernmost squares in the east, (b) the 10 northernmost squares in the west, and (c) the 10 squares furthest inland. We used only well‐recorded squares for the two newer time periods, but all squares for 1901–2000. Violin plots show the estimated kernel probability density (the width of the violins shows the proportion of the data at the *y*‐axis values), and boxes show the median as well as 25th and 75th percentiles of the data. Circles show all data points. Letters inside the graphs indicate significant differences in pairwise comparisons (Tukey tests); time periods with shared letters do not differ significantly from each other. We report complete statistics from the Tukey tests in Table S6.

*Lasiommata megera* has expanded both northwards and towards the Swedish inland (Figure 2), and the expansion coincides with regional climatic warming (compare Figure 2 with Figure 3). The 10 northernmost sightings were reported further north in recent time periods than in 1901–2000 both in the north‐east (*F*_2,27_ = 15.57, *p* < 0.001; Figure 4a) and north‐west (*F*_2,27_ = 19.47, *p* < 0.001; Figure 4b). The 10 observations furthest from their nearest coastline were reported further inland in the recent time periods than in 1901–2000 (*F*_2,27_ = 12.76, *p* < 0.001; Figure 4c).

### Diapause induction experiments

**FIGURE 5:**
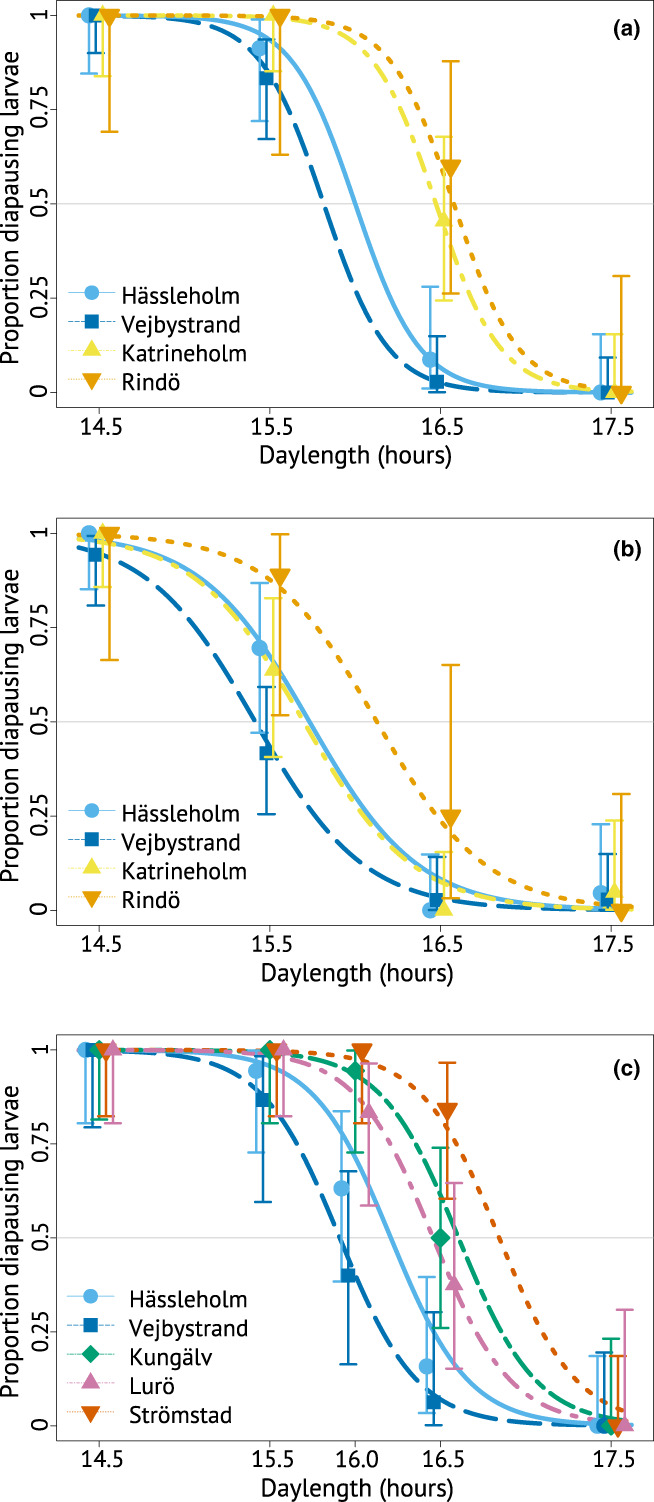
Diapause decision in response to daylength. (a) Eastern cline, 16°C; (b) eastern cline, 22°C; (c) western cline, 16°C. Curves show fitted probabilities from our models, symbols show actual proportions of individuals that entered diapause in each treatment and population, and vertical lines represent 95% confidence intervals. The grey horizontal line highlights 50% diapause; the critical daylengths are the *x* values at which the curves cross the grey line.

Daylength had, by far, the largest effect on diapause induction in both the eastern (*χ* ^2^ = 652.89, df = 1, *p* < 0.001) and western cline experiment (*χ* ^2^ = 339.32, df = 1, *p* < 0.001). Caterpillars from all populations were more likely to enter diapause in short than in long daylengths. In the eastern cline experiment, we found an effect of temperature. Fewer caterpillars entered diapause in 22°C than in 16°C (*χ* ^2^ = 36.39, df = 1, *p* < 0.001), but there was no interaction between temperature and population (*χ* ^2^ = 4.32, df = 3, *p* = 0.23), that is, the temperature effect did not differ among populations. Populations differed in their responses to daylength in both experiments (eastern cline: *χ* ^2^ = 20.16, df = 3, *p* < 0.001; western cline: *χ* ^2^ = 36.48, df = 4, *p* < 0.001). Logistic regression curves show that caterpillars from northern populations were more likely than those from southern populations to enter diapause in long daylengths (Figure 5), and the effect of temperature is seen when comparing curves between Figures 5a and 5b. Population did not interact with daylength, that is, daylength reaction norms take similar shapes in all populations (eastern cline: *χ* ^2^ = 0.61, df = 3, *p* = 0.90; western cline: *χ* ^2^ = 6.41, df = 4, *p* = 0.17). Our alternative models with different thresholds for scoring diapause and non‐diapause gave results similar to the models reported here (Table S7). Family (the random effect) did not explain variation in either the eastern (estimated variance = 1.281e‐12; adjusted *p* = 0.50) or western cline experiment (estimated variance = 0; adjusted *p* = 0.50).

We report full statistics from pairwise comparisons of all pairs of populations in Table S8. In 16°C, the north‐eastern populations Katrineholm and Rindö differed significantly from the southern populations (Katrineholm–Hässleholm, *p* = 0.037; Katrineholm–Vejbystrand, *p* = 0.002; Rindö–Hässleholm, *p* = 0.023; Rindö–Vejbystrand, *p* = 0.002). In 22°C, only the difference between Rindö and Vejbystrand was significant (*p* = 0.022). Along the western cline, the northern population Strömstad differed significantly from both Hässleholm and Vejbystrand (*p* < 0.001 for both), but the other northern population, Lurö, differed significantly only from Vejbystrand (*p* < 0.001). The intermediate population Kungälv differed significantly from both southern populations (Hässleholm, *p* = 0.017; Vejbystrand, *p* < 0.001), but not from the northern ones.

**FIGURE 6:**
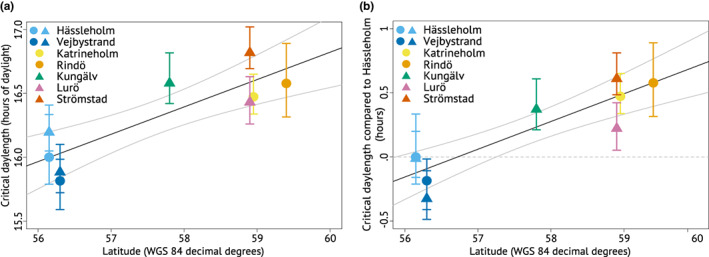
Critical daylength estimates with 95% confidence intervals in both 16°C treatments. Circles show the populations in the eastern cline populations, and triangles the populations in the western cline experiment. Hässleholm and Vejbystrand have two symbols because they were used in both experiments. (a) Estimated critical daylengths plotted against latitude (regression: *Z* = 4.1265, *p* < 0.001, *R* ^2^ = 0.71). (b) Difference in critical daylength from the critical daylength estimate (obtained from each respective experiment) for the southernmost population, Hässleholm (regression: *Z* = 5.0611, *p* < 0.001, *R* ^2^ = 0.82). The black regression lines and the grey curves indicating their 95% confidence limits are based on predicted values from linear models fitted with the rma.uni function in R package metafor 3.0.2 (Viechtbauer, 2010). This method accounts for the bootstrapped uncertainties for each individual critical daylength.

Critical daylengths in 16°C along both clines are shown in Figure 6. The northern populations, except Lurö, had 28–56 min longer critical daylengths than the southernmost population, Hässleholm. The western cline experiment generally produced longer critical daylength estimates than the eastern cline experiment. To aid comparison between the experiments, we correct for this in Figure 6b, where each population's critical daylength is compared to that estimated for Hässleholm in the same experiment. We report all critical daylength estimates, including those for 22°C in the eastern cline experiment, in Table S9.

## DISCUSSION

We demonstrate parallel evolution to the new daylength conditions that organisms face when expanding polewards under climate warming. Three out of four *L. megera* populations near the fronts of two independent northward range expansions have rapidly evolved local adaptation despite theory suggesting genetic constraints to adaptation at range margins. One north‐western population and the intermediate‐latitude population had intermediate phenotypes between those of the southern populations and the other north‐western population. Citizen science data show that *L. megera* has expanded northwards and towards the inland over the last 20 years and that our northern populations are at or near the current range edge. The range expansion appears to have followed recent climate warming, and our study is the first to demonstrate local adaptation to daylength cues during a range expansion most likely driven by anthropogenic climate change.

Rapid adaptive evolution of critical daylength during range expansion has previously been shown in an invasive mosquito, which has expanded across 15 latitudinal degrees in the United States; it displayed local adaptation to daylength 23 years after its introduction (Urbanski et al., 2012). Range expansions of native and invasive species can have different evolutionary dynamics (Colautti & Lau, 2015; Moran & Alexander, 2014), but we show that responses to daylength cues have evolved rapidly also during range expansion of a native species with a continuous range. Yet other studies have revealed that insects' responses to daylength cues can evolve in just 5 years (Bean et al., 2012; Bradshaw & Holzapfel, 2001), but no range expansion (other than initial introductions) was involved in those works. Taken together, these studies represent growing evidence that the need to evolve responses to unfamiliar daylength cues need not prevent range expansions.

Although we cannot know what critical daylengths perfectly adapted populations would have, our critical daylength estimates signal accurate local adaptation. Our estimates match classic studies showing critical daylengths within various insect species to differ by about 60–90 min per five latitudinal degrees (Danilevsky et al., 1970; Tauber et al., 1986, pp. 201–207). Extrapolating from our data, the critical daylengths of the northern populations, except Lurö, would be 50–70 min longer per five latitudinal degrees, if compared to Hässleholm, and 73–110 min longer, if compared to Vejbystrand. Lurö, however, showed an intermediate response, differing significantly from Vejbystrand, but no other populations. There is no indication that Lurö would be warmer than the coastal areas Strömstad and Rindö (compare Figures 1 and 3), whose populations showed longer critical daylengths than Lurö's. Instead, a plausible explanation—that would have to be tested with genetic data—for Lurö's response is the population's geographic isolation on islands in a major lake (Figures 1 and 2). Gene flow from the south would require *L. megera* to cross at least 10 km of open water, and no populations are known close to this shortest route. Gene flow from the north, in turn, seems even more unlikely because there are no known stable populations in this direction. Given this geographic isolation, founder effects are likely to influence Lurö more than the other northern range margin populations; the founders of the latter are likely to have come from neighbouring populations and thus from further north and in greater numbers than the rare long‐range migrants that would have founded Lurö.

Another notable result is that the western cline experiment produced longer critical daylength estimates than the eastern cline experiment (Figure 6). Besides chance and uncontrolled differences in rearing conditions, epigenetic effects could contribute to this result. The caterpillars in the western cline experiment were F1 offspring of females caught in late summer and would in natural conditions mostly enter diapause, but the caterpillars in the eastern cline experiment were offspring of hibernated individuals (whose mothers were field‐caught) and would in natural conditions develop without diapause. However, our results speak against substantial maternal effects on diapause induction. Both experiments yielded similar results, including for the two southernmost populations that we used in both experiments and for which both F1 and F2 offspring of wild‐caught females were tested. Moreover, the magnitude with which both spring and autumn offspring from all populations responded to daylength treatments contrasts with results from species with described maternal effects. In such species, maternal effects cannot be overridden as completely in the diapausing life stage (Huestis & Marshall, 2006; Kogure, 1933; Mousseau & Dingle, 1991). We recommend caution in comparing critical daylength estimates among experiments, but as the two southern populations differed in the same direction in both experiments, the overall difference between our experiments should not change the interpretation of our results.

The local adaptation that we report should be viewed in the light of the extensive, but not widely tested, theoretical literature about genetic processes that can hinder local adaptation in range margin populations. Given the continuous range of *L. megera* along the Swedish coasts, some of our study populations could receive much gene flow and suffer from swamping by locally maladaptive alleles from the south. These well‐connected northern populations nevertheless seem locally adapted. Evidence from works where genetics have been studied suggest, on the one hand, that strong gene flow does not necessarily prevent local adaptation at range margins (Gonzalo‐Turpin & Hazard, 2009; Muir et al., 2014; Tyukmaeva et al., 2011) and, on the other hand, that critical daylength can evolve despite the bottlenecks and founder effects that are likely in expanding populations (Bean et al., 2012). At the same time, the geographically isolated Lurö population might represent a case where genetic constraints, such as founder effects, low genetic variation, or genetic drift have slowed local adaptation down.

Daylength largely dictated the developmental pathways of caterpillars, but caterpillars in 16°C entered diapause in longer daylengths than those in 22°C. Temperature modifies daylength responses in many other insects too (Tauber et al., 1986, pp. 112–160). The adaptive significance of this effect in populations facing climate warming could be two‐fold. First, being sensitive to temperature may help populations modify their diapause decisions in exceptional years, for example, by developing an additional generation during warm autumns. Second, the effect of temperature may be beneficial in populations that are not yet well adapted to the daylengths that they experience, if cold temperatures lengthen maladaptively short critical daylengths when needed. Such plasticity could help explain the short‐term success (Chen et al., 2011; Parmesan & Yohe, 2003) of many insect species that have rapidly moved northwards.

Voltinism (the number of adult generations per year) further complicates the relationship between critical daylength and latitude. In *Pararge aegeria* (Nymphalidae: Satyrinae, the speckled wood), critical daylengths differed by 2.4 h—compared to only 1–2 h in our *L. megera* study—over a similar south–north cline in Sweden (Lindestad et al., 2019). Unlike *L. megera*, *P. aegeria* switches from bivoltinism to univoltinism within this geographic range. Northern, univoltine populations enter diapause in the long days of July, whereas southern, bivoltine populations develop directly into adults under such conditions. In these bivoltine populations, diapause is not induced before the short days of August. This way, a voltinism shift explains the large geographic differences in *P. aegeria* critical daylengths. Regarding *L. megera*, an intriguing question arises: could *L. megera* also switch to univoltinism at high latitudes? Such a shift would be needed for the species to thrive wherever summers are too short to support a bivoltine life cycle, but the shift would require much longer critical daylengths than we estimated for any population. Producing more generations than season length can support can lead to a ‘lost generation’ (Van Dyck et al., 2015). This phenomenon may contribute to the recent decline of *L. megera* in Western Europe, where climate warming has pushed formerly bivoltine populations into growing a third generation—one that might not make it into the diapausing stage before winter (Van Dyck et al., 2015).

Range shifts and adaptation are important ways for organisms to cope with climate change (Parmesan, 2006), and these processes should be considered together. Species may benefit from changing both their ranges and their phenology (Hällfors et al., 2021), and range‐expanding (or range‐shifting) populations need to adapt to conditions—such as daylength—that do not change with the climate. The ability of species to adapt during range expansions influences which species can establish in new areas and which species cannot, and the importance of evolution is rightfully gaining recognition in models for predicting species distributions (Diamond, 2018). We have shown rapid adaptation to new daylength regimes during poleward range expansion, but also that factors such as temperature effects and voltinism should be considered when predicting where a species could establish and thrive. Field studies assessing how daylength and temperature affect diapause induction and, ultimately, fitness at range margins and in potential future environments of species would be valuable additions to our work. Genetic studies, in turn, can clarify the role of genetic constraints to local adaptation in expanding populations. Furthermore, future studies should address how our results hold for other species—including animals and plants with longer generation times—and other traits; is the apparent ease with which responses to daylength have evolved in both our study system and other insects (Bean et al., 2012; Bradshaw & Holzapfel, 2001; Gomi, 2007; Urbanski et al., 2012) the rule or an exception?

Our results, implying that latitudinal differences in daylength need not strongly limit climate‐induced range expansions, may be bad news to people concerned about the spread of disease vectors or agricultural and forest pests, but good news to endangered species. However, if Lurö is poorly adapted (as our results suggest) and genetically isolated, our results would also indicate that local adaptation might fail in isolated or small populations—the kind of populations typical for endangered species (Frankham, 2005; Shaffer, 1981). For management resources to be allocated well, scientists must be able to predict future ranges of various species with disparate life histories and population genetic backgrounds. Such prediction requires knowledge, from many different species, about what ecological and genetic circumstances allow expanding range margin populations to adapt (Diamond, 2018; Forrest, 2016; Lehmann et al., 2014, 2020; Saikkonen et al., 2012). In this study, we have taken essential steps towards understanding whether, how, and how fast expanding populations can adapt to new environments—some of the big questions that ecologists and evolutionary biologists must try to answer in a time shaped by rapid climate change.

## AUTHOR CONTRIBUTIONS

Karl Gotthard and Mats Ittonen conceived and planned the study. All authors collected wild butterflies for the experiments. Mats Ittonen and Alexandra Hagelin performed the experiments and analysed the experimental data. Mats Ittonen compiled and analysed the species occurrence data and drafted the manuscript. All authors revised drafts and accepted the final version of the manuscript.

### PEER REVIEW

The peer review history for this article is available at https://publons.com/publon/10.1111/ele.14085.

### OPEN RESEARCH BADGES

This article has earned an Open Data badge for making publicly available the digitally‐shareable data necessary to reproduce the reported results. The data is available at [https://doi.org/10.5061/dryad.4qrfj6qd2].

## Supporting information


Appendix S1

## Data Availability

The data used in this study are available at https://doi.org/10.5061/dryad.4qrfj6qd2. The R script is available at https://doi.org/10.5281/zenodo.6891471.
