# Supplementary material for: Local adaptation to seasonal cues at the fronts of two parallel, climate‐induced butterfly range expansions
Source: Ecol Lett. 2022 Aug 15;25(9):2022–33. doi: 10.1111/ele.14085 (PMC9544862; doi:10.1111/ele.14085)
Supplement: Supplementary file 1 — Appendix S1 [file ELE-25-2022-s001.pdf]

Supporting information for

Ittonen, M., Hagelin, A., Wiklund, C. & Gotthard, K. (2022). Local adaptation to seasonal cues at the fronts of two parallel, climate-induced butterfly range expansions. *Ecology Letters*

This document includes Tables S1–S9 and Figures S1 and S2

Table S1. Numbers of reported observations of Papilionoidea butterflies, species of Papilionoidea butterflies, and *Lasiommata megera* by time period for each country that we used data from.

Country	Time period	Papilionoidea observations	Papilionoidea species	*L. megera* observations
Sweden	1901–2000	40,265	167	266
Sweden	2001–2010	344,940	123	2,614
Sweden	2011–2020	723,956	131	6,234
Norway	1901–2000	24,505	116	79
Norway	2001–2010	24,111	108	213
Norway	2011–2020	121,058	110	1,018
Finland	1901–2000	424,833	113	7
Finland	2001–2010	277,803	104	5
Finland	2011–2020	390,635	113	2
Denmark	1901–2000	243,760	100	3,111
Denmark	2001–2010	102,570	84	788
Denmark	2011–2020	197,803	88	1,682
Lithuania	1901–2000	8,515	116	251
Lithuania	2001–2010	11,580	115	27
Lithuania	2011–2020	22,273	120	188
Latvia	1901–2000	170	56	0
Latvia	2001–2010	30	23	0
Latvia	2011–2020	309	71	7
Estonia	1901–2000	17,951	125	0
Estonia	2001–2010	16,389	107	0
Estonia	2011–2020	67,549	118	2

Table S2. Coordinates (WGS 84 decimal degrees) for, sizes of, and distances between sampling locations. We collected butterflies from 1–5 habitat patches within each population. We calculated the areas of the habitat patches and distances between them using the *Measure distance and area* tool in Google Earth version 9.164.0.1 - WebAssembly; (https://earth.google.com/web/, Accessed 16 June 2022). The maximum distance between patches is the shortest distance (straight line) between the patches furthest apart. The coordinates correspond to the point in the middle of this shortest line between the patches furthest apart.

Population	Coordinates	Sizes of habitat patches (km2)	Maximum distance between patches (km)
Hässleholm	56.15 13.73	0.009; 0.012; 0.025; 0.046; 0.067	10.6
Vejbystrand	56.3, 12.8	0.166	
Katrineholm	58.95, 16.27	0.101; 0.061; 0.089	8.8
Rindö	59.40,18.40	0.012; 0.016	3.9
Kungälv	57.82, 11.91	0.014; 0.018; 0.078; 0.091	5.6
Lurö	58.86, 13.24	0.015; 0.054	2.5
Strömstad	58.85, 11.15	0.0005; 0.018; 0.025	2.0

Table S3. Measured mean temperatures ± one standard deviation in each of our experimental treatments.

Cline	Set temperature	Daylength	Measured mean temperature ± SD
Eastern	16	14.5	15.83 ± 0.35
Eastern	16	15.5	15.87 ± 0.25
Eastern	16	16.5	15.55 ± 0.45
Eastern	16	17.5	15.11 ± 0.31
Eastern	22	14.5	21.60 ± 0.41
Eastern	22	15.5	22.31 ± 0.50
Eastern	22	16.5	22.04 ± 0.39
Eastern	22	17.5	22.13 ± 0.56
Western	16	14.5	16.62 ± 0.36
Western	16	15.5	16.69 ± 0.49
Western	16	16.0	16.02 ± 0.25
Western	16	16.5	16.17 ± 0.37
Western	16	17.5	16.57 ± 0.15

Table S4. Sample sizes and mortality per treatment and population in the eastern cline experiment. The first numbers are the sample sizes after mortality, the numbers in parentheses are the numbers of mothers contributing to the sample, and the numbers in square brackets are the numbers of caterpillars that died before the end of the experiment.

Temperature (°C)	Daylength (h)	Hässleholm	Vejbystrand	Katrineholm	Rindö
16	14.5	22 (6) [2]	35 (9) [1]	21 (4) [2]	10 (2) [1]
16	15.5	23 (6) [1]	36 (9) [0]	23 (4) [0]	8 (2) [3]
16	16.5	23 (6) [1]	35 (9) [1]	22 (4) [1]	10 (2) [1]
16	17.5	22 (6) [3]	38 (9) [3]	22 (4) [1]	10 (2) [1]
22	14.5	23 (6) [1]	35 (9) [1]	24 (4) [0]	9 (2) [2]
22	15.5	23 (6) [1]	36 (9) [0]	22 (4) [1]	9 (2) [1]
22	16.5	23 (6) [1]	37 (9) [0]	22 (4) [1]	8 (2) [3]
22	17.5	22 (6) [2]	35 (9) [1]	21 (3) [2]	10 (2) [1]

Table S5. Sample sizes and mortality per treatment and population in the western cline experiment. The first numbers are the sample sizes after mortality, the numbers in parentheses are the numbers of mothers contributing to the sample, and the numbers in square brackets are the numbers of caterpillars that died before the end of the experiment.

Daylength (h)	Hässleholm	Vejbystrand	Kungälv	Lurö	Strömstad
14.5	17 (6) [3]	16 (5) [4]	18 (5) [2]	17 (7) [3]	19 (9) [1]
15.5	18 (6) [2]	15 (5) [5]	17 (6) [2]	19 (7) [1]	19 (9) [1]
16	19 (7) [1]	15 (5) [5]	18 (6) [2]	18 (7) [1]	17 (9) [3]
16.5	19 (7) [1]	16 (5) [3]	18 (5) [1]	16 (7) [2]	19 (9) [1]
17.5	18 (7) [2]	17(5) [3]	14 (5) [5]	18 (7) [2]	17 (9) [2]

Table S6. Results from pairwise comparisons (Tukey tests) between time periods based on our linear models, with which we analysed how time periods differ in the latitudes of the northernmost observations east of 14.31 °E, the latitudes of the northernmost observations west of 14.31 °E, and the distances from the nearest coastlines. The latitudinal analysis was made using coordinates in the SWEREF 99 coordinate reference system, and the analysis of distance to coast was made with ln-transformed data. Hence, the estimated means are not directly comparable with Figure 4 in the article.

Model	Comparison	Estimated difference in mean ± SE	t	P
Eastern, northernmost observations	1901–2000 *vs* 2001–2010	−42,000 ± 15,415	−2.73	0.029
Eastern, northernmost observations	1901–2010 *vs* 2011–2020	−86,000 ± 15,415	−5.58	<0.001
Eastern, northernmost observations	2001–2010 *vs* 2011–2020	−44,000 ± 15,415	−2.85	0.022
Western, northernmost observations	1901–2000 *vs* 2001–2010	−46,000 ± 9,813	−4.69	<0.001
Western, northernmost observations	1901–2010 *vs* 2011–2020	−58,000 ± 9,813	−5.91	<0.001
Western, northernmost observations	2001–2010 *vs* 2011–2020	−12,000 ± 9,813	−1.22	0.45
Distance from coastline	1901–2000 *vs* 2001–2010	−0.19 ± 0.14	−1.36	0.37
Distance from coastline	1901–2010 *vs* 2011–2020	−0.69 ± 0.14	−4.90	<0.001
Distance from coastline	2001–2010 *vs* 2011–2020	−0.50 ± 0.14	−3.53	0.004

Table S7. Likelihood ratio test results for generalized linear mixed models with diapause/non-diapause scored after alternative numbers of days from the start of the experiment. Shorter = individuals not pupated earlier than 49 (16 °C treatments) or 29 days (22 °C treatments) after the start of the experiment were scored as diapausing. Longer = individuals not pupated earlier than 61 (16 °C treatments) or 37 days (22 °C treatments) after the start of the experiment were scored as diapausing.

**Eastern cline experiment**				
**Alternative threshold**	**Variable**	**χ^2**	**df**	**p**
Shorter	Daylength	530.50	1	< 0.001
Shorter	Temperature	46.05	3	< 0.001
Shorter	Population	19.39	3	< 0.001
Shorter	Temperature × population	1.91	3	0.59
Shorter	Daylength × population	2.39	3	0.50
Longer	Results with the longer thresholds were identical to those of the model reported in the article.			
**Western cline experiment**				
**Alternative threshold**	**Variable**	**χ^2**	**df**	**p**
Shorter	Daylength	329.02	1	< 0.001
Shorter	population	23.53	4	< 0.001
Shorter	Daylength × population	8.28	4	0.082
Longer	Daylength	366.95	1	< 0.001
Longer	population	43.03	4	< 0.001
Longer	Daylength × population	2.22	4	0.70

Table S8. Results from Tukey tests comparing the diapause incidence between populations. Differences that are significant (with the threshold value of 0.05) are shown in bold.

Experiment	Temperature	Comparison	Difference between predictions ± SE	Z	P
Eastern	16	Hässleholm–Vejbystrand	0.98 ± 0.79	−1.25	0.59
Eastern	16	Hässleholm–Katrineholm	−2.52 ± 0.94	−2.67	**0.037**
Eastern	16	Hässleholm–Rindö	−3.08 ± 1.08	−2.84	**0.022**
Eastern	16	Vejbystrand–Katrineholm	−3.50 ± 1.00	−3.52	**0.002**
Eastern	16	Vejbystrand–Rindö	−4.06 ± 1.13	−3.52	**0.002**
Eastern	16	Katrineholm–Rindö	−0.56 ± 0.87	−0.64	0.91
Eastern	22	Hässleholm–Vejbystrand	0.99 ± 0.52	1.90	0.22
Eastern	22	Hässleholm–Katrineholm	0.11 ± 0.61	0.17	>0.99
Eastern	22	Hässleholm–Rindö	−1.24 ± 0.80	−1.54	0.40
Eastern	22	Vejbystrand–Katrineholm	−0.89 ± 0.57	-1.56	0.39
Eastern	22	Vejbystrand–Rindö	−2.23 ± 0.78	−2.85	**0.022**
Eastern	22	Katrineholm–Rindö	−1.34 ± 0.83	−1.61	0.37
Western	16	Hässleholm–Vejbystrand	1.31 ± 0.54	2.43	0.11
Western	16	Hässleholm–Kungälv	−1.74 ± 0.56	3.09	**0.017**
Western	16	Hässleholm–Lurö	−1.09 ± 0.54	2.01	0.26
Western	16	Hässleholm–Strömstad	−2.78 ± 0.65	−4.27	**<0.001**
Western	16	Vejbystrand–Kungälv	−3.05 ± 0.62	−4.91	**<0.001**
Western	16	Vejbystrand–Lurö	−2.40 ± 0.59	−4.05	**<0.001**
Western	16	Vejbystrand–Strömstad	−4.09 ± 0.72	−5.68	**<0.001**
Western	16	Kungälv–Lurö	0.65 ± 0.58	−1.13	0.79
Western	16	Kungälv–Strömstad	−1.04 ± 0.65	−1.60	0.49
Western	16	Lurö–Strömstad	−1.70 ± 0.65	−2.61	0.068

Table S9. Critical daylength estimates and their bootstrapped 95 % confidence intervals. Units: WGS 84 decimal degrees for latitude and longitude, °C for temperature, and hours for critical daylength.

Experiment	Population	Latitude	Longitude	Temperature	Critical daylength	Confidence interval
Eastern	Hässleholm	56.15	13.73	16	16.00	15.78–16.34
Eastern	Vejbystrand	56.30	12.80	16	15.82	15.59–15.99
Eastern	Katrineholm	58.95	16.25	16	16.47	16.33–16.65
Eastern	Rindö	59.40	18.40	16	16.58	16.32–16.89
Eastern	Hässleholm	56.15	13.73	22	15.73	15.53–15.95
Eastern	Vejbystrand	56.30	12.80	22	15.41	15.24–15.60
Eastern	Katrineholm	58.95	16.25	22	15.70	15.48–16.03
Eastern	Rindö	59.40	18.40	22	16.13	15.76–16.45
Western	Hässleholm	56.15	13.7	16	16.21	16.05–16.41
Western	Vejbystrand	56.30	12.80	16	15.90	15.72–16.10
Western	Kungälv	57.82	11.91	16	16.59	16.42–16.82
Western	Lurö	58.86	13.24	16	16.44	16.26–16.63
Western	Strömstad	58.85	11.15	16	16.83	16.69–17.02

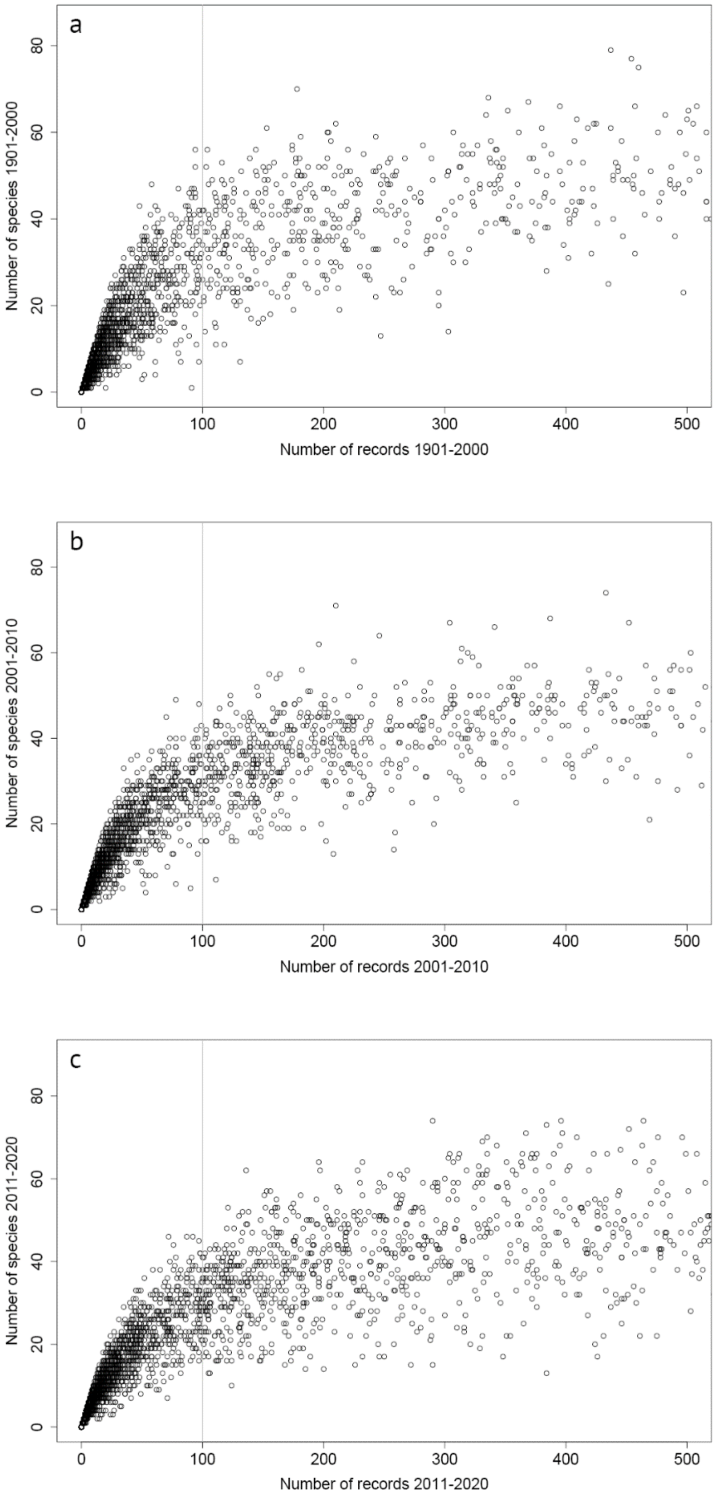

Figure S1. The relationship between number of observations and number of species of Papilionoidea butterflies recorded from within each 20×20 km map square (shown as circles) in the time periods 1901–2000 (a), 2001–2010 (b), and 2011–2020 (c). The grey vertical line highlights 100 observations, which was our threshold for considering a square well-recorded.

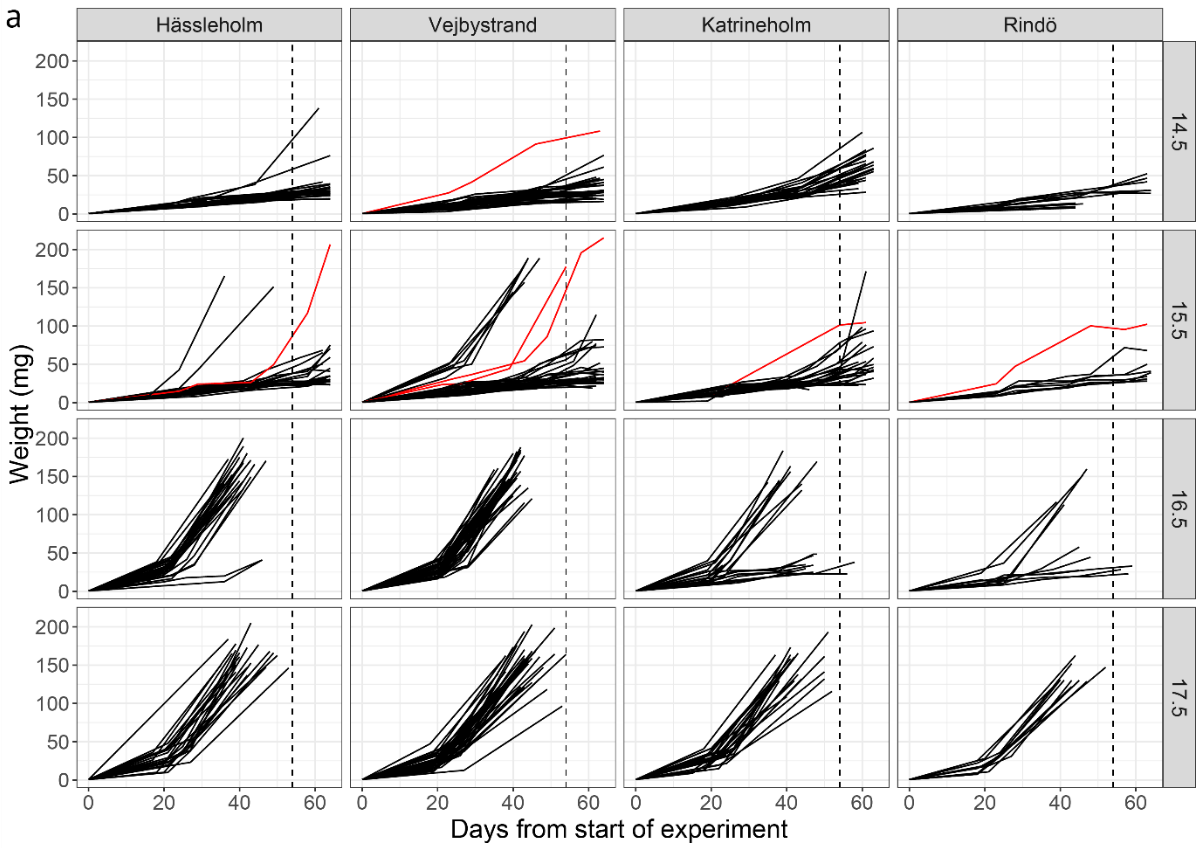
a
Hässleholm
Vejbystrand
Katrineholm
Rindö
14.5
15.5
16.5
17.5
Weight (mg)
Days from start of experiment

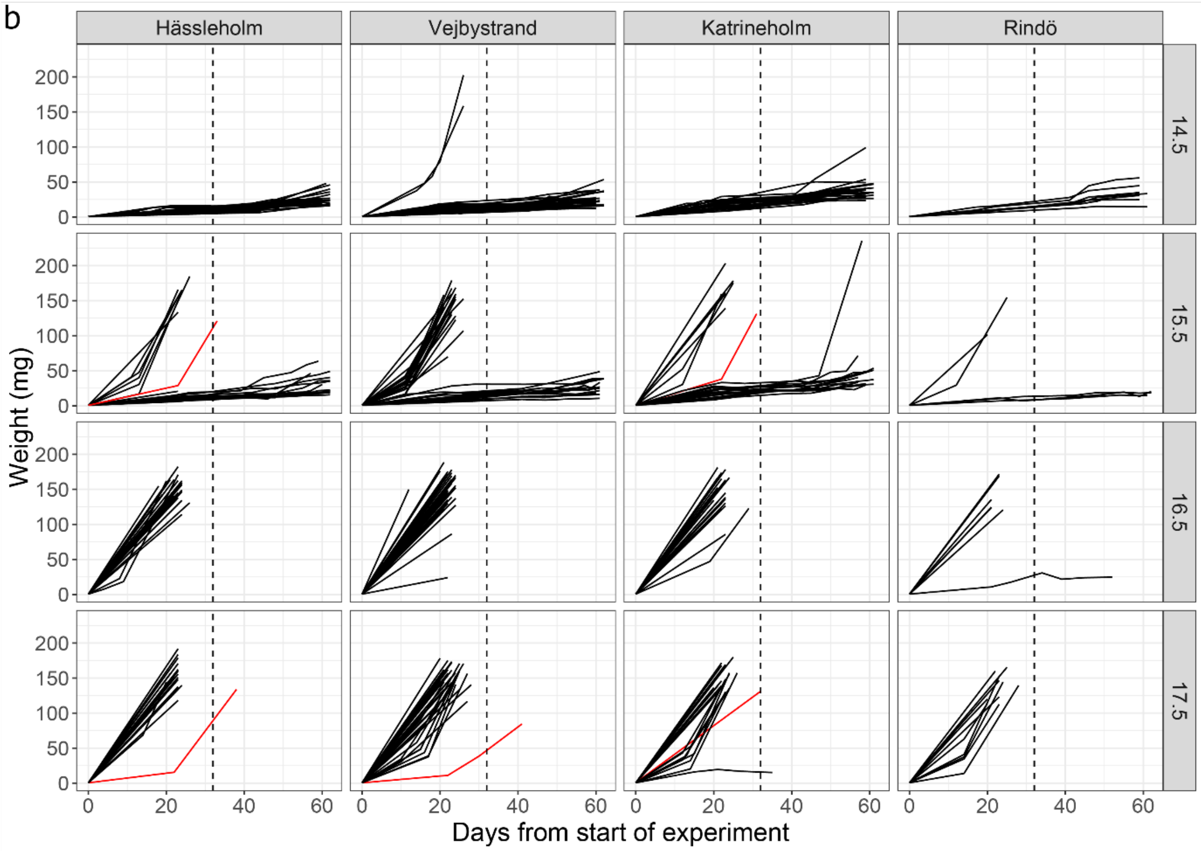
b
Hässleholm
Vejbystrand
Katrineholm
Rindö
14.5
15.5
16.5
17.5
Weight (mg)
Days from start of experiment

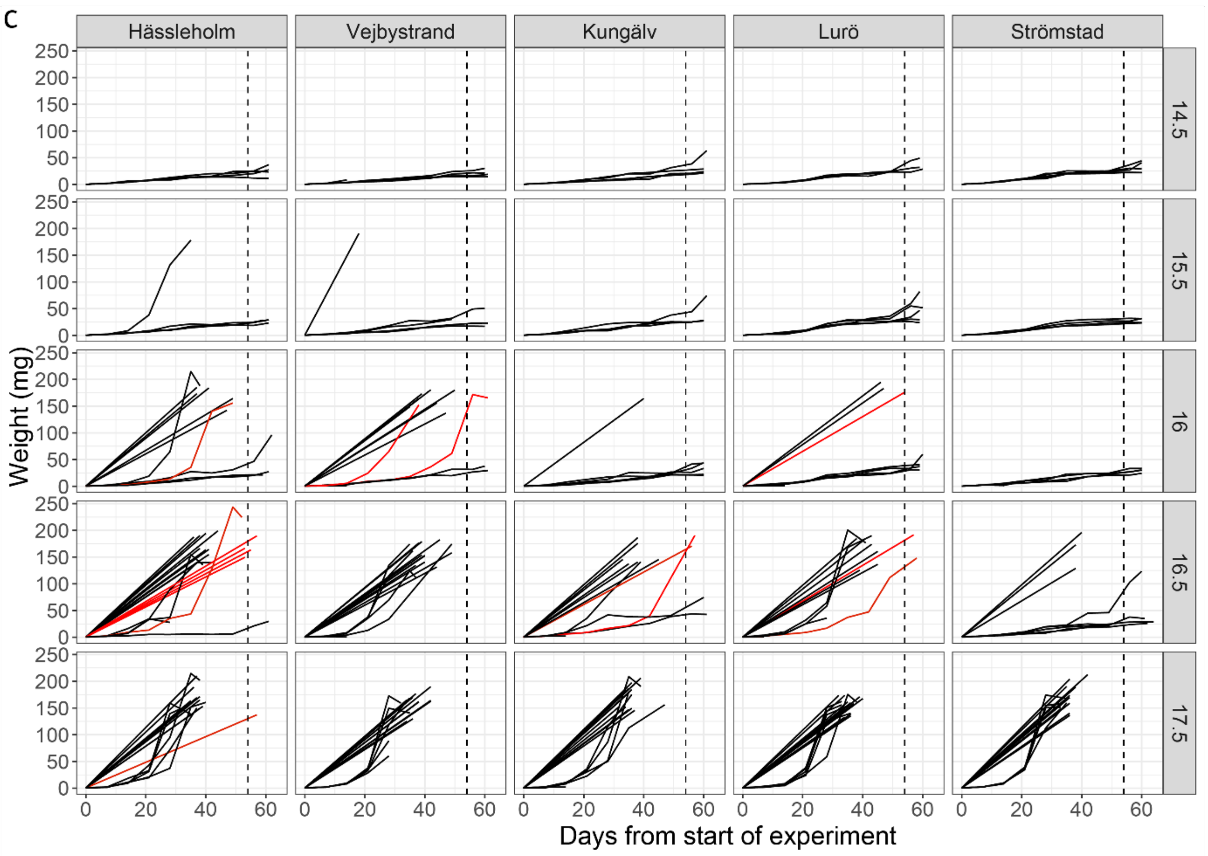

Figure S2. Growth curves of individual caterpillars in (a) the 16 °C treatment in the eastern cline experiment, (b) the 22 °C treatment in the eastern cline experiment, and (c) the western cline experiment (16 °C). Each panel shows one population in one treatment and each curve shows the weight development of one individual. The vertical dashed lines show the threshold number of days for defining direct development and diapause. Individuals with curves that end at or to the left of the dashed line were scored as non-diapausing, and those with curves continuing to the right of the dashed line were scored as diapausing. Red curves highlight the few individuals that we, based on comparing with the shapes of the curves of clearly diapausing or directly developing individuals, think could reasonably have been considered either diapausing or directly developing. We weighed caterpillars in the eastern cline experiment (a, b) only after they reached their third larval instar. During the western cline experiment (c), we weighed only a subset of caterpillars, but did that with one-week intervals throughout the experiment. For the individuals in (c) that we did not weigh as caterpillars, the curves only connect the average start weight of those individuals that were weighed with the measured pupal weight. Hence, the growth curves from the western cline experiment cannot be as directly compared as those from the eastern cline experiment.
